# A novel multi-DoF surgical robotic system for brachytherapy on liver tumor: Design and control

**DOI:** 10.1007/s11548-021-02380-7

**Published:** 2021-05-01

**Authors:** Xiaofeng Lin, Shoujun Zhou, Tiexiang Wen, Shenghao Jiang, Cheng Wang, Jingtao Chen

**Affiliations:** 1 Shenzhen Institute of Advanced Technology, Chinese Academy of Sciences, 1068 Xueyuan Avenue, Shenzhen University Town, Shenzhen, GD 518055 People’s Republic of China; 2 University of Chinese Academy of Sciences, Beijing, People’s Republic of China; 3 National Innovation Center for Advanced Medical Devices, Shenzhen, GD 518110 People’s Republic of China

**Keywords:** Robotics, Brachytherapy, Artificial potential field, Motion planning

## Abstract

****Purpose**:**

Radioactive seed implantation is an effective invasive treatment method for malignant liver tumors in hepatocellular carcinomas. However, challenges of the manual procedure may degrade the efficacy of the technique, such as the high accuracy requirement and radiation exposure to the surgeons. This paper aims to develop a robotic system and its control methods for assisting surgeons on the treatment.

****Method**:**

We present an interventional robotic system, which consists of a 5 Degree-of-Freedom (DoF) positioning robotic arm (a 3-DoF translational joint and a 2-DoF revolute joint) and a needle actuator used for needle insertion and radioactive seeds implantation. Control strategy is designed for the system to ensure the safety of the motion. In the designed framework, an artificial potential field (APF)-based motion planning and an ultrasound (US) image-based contacting methods are proposed for the control.

****Result**:**

Experiments were performed to evaluate position and orientation accuracy as well as validate the motion planning procedure of the system. The mean and standard deviation of targeting error is 0.69 mm and 0.33 mm, respectively. Needle placement accuracy is 1.10 mm by mean. The feasibility of the control strategy, including path planning and the contacting methods, is demonstrated by simulation and experiments based on an abdominal phantom.

****Conclusion**:**

This paper presents a robotic system with force and US image feedback in assisting surgeons performing brachytherapy on liver tumors. The proposed robotic system is capable of executing an accurate needle insertion task with by optical tracking. The proposed methods improve the safety of the robot’s motion and automate the process of US probe contacting under the feedback of US-image.

## Introduction

Liver cancer has been regarded as the 6th most commonly diagnosed cancer and 4th leading cause of cancer death worldwide in 2018. The bulk of primary liver cancer is hepatocellular carcinomas (HCC), which comprises 75% to 85% of cases [1]. Recently, 125-iodine seed implantation shows promising therapeutic effects on the treatment of malignant liver tumor in HCC [2]. It can release low-dosage radiation and increase the dosage distribution ratio between locally radioactive tissues and normal tissues, which has also been proven to have advantages in relieving short-term pain and improving the quality of life for patients [3].

**Fig. 1:**
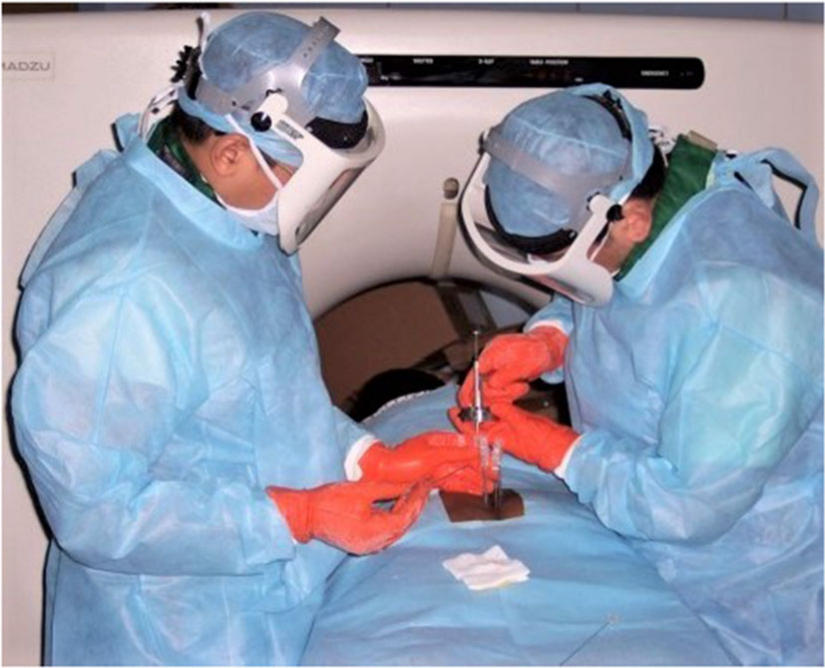
Radioactive seeds implantation in HCC

In a traditional application, performing brachytherapy requires high-accuracy seeds placement, which is biologically and clinically acceptable with an accuracy of 2 mm [4]. However, the accuracy of seeds implantation, which is vital for the efficacy of the technique, is highly dependent upon surgeons’ experience, especially for large tumors. A research proposed by Podder et al. [5] shows that manual placement techniques (Fig. 1) have an estimated in vivo accuracy of 3–6 mm, suggesting the demand for a robotic device with high accuracy to assist operation. Besides, radiation from seeds limits the time that a surgeon could spend in executing the implantation manually [5]. Motorized implantation devices can also reduce radiation exposure to medical staff and prolong the essential time to perform high-accuracy implantation. It’s also an underlying problem with the movement of the liver caused by breathing, which makes the tumors tracking difficult. To stabilize needle manipulation, as well as increasing the accuracy, dexterity and repeatability of the surgery, surgical robots with medical image guidance may be better candidates for the implantation of radioactive seeds [6].

Recently, several research groups have proposed their works on robot-assisted brachytherapy. Zequn et al. [7] developed a robotic system for brachytherapy with 7-DoF manipulator KUKA iiwa 14 (KUKA, Germany) and seeds implantation end tool. The system is equipped with force/optical feedback and has a systematic strategy for calibrating the robotic system. Zhu et al. [8] put forward a novel 6-DoF arch-like structure for needle orientation, with an end effector of seeds’ delivering on it. The system had its path planning and needle positioning by preoperative computerized tomography (CT) scanning, and its motion-tracking by an optical tracking system (OTS).

However, the frameworks of Zequn et al. and Zhu et al. are short of real-time feedback from patients, which makes it difficult to monitor the real-time position of the tumor in surgery. Jiang et al. [9] and Cunha et al. [10] integrated real-time US or magnetic resonance (MR) image guidance into their work. Nevertheless, their systems are based on brachytherapy for the prostate, which may be hard applying in the therapy of liver tumors because of the requirement of workspace or DoF. A robotic system for brachytherapy on liver tumors requires both accurate seed placement and a large enough workspace regarding the size of the liver. Besides, US feedback is necessary considering the dynamic environment of the abdomen, where the end effector of the robot move close to patients and contact with them. It may cause unexpected accidents such as collision and rubbing between the probe and the patient on the trajectory. Also, because of the dynamic contacting process on the abdomen, it is hard to decide the goal of an end effector with the US probe. The position where the probe can acquire high-quality US images is undefined before the operation. Surgeons may need to manually stop the robot in this process without computer assistance.

The goal of our study is to develop a robotic system and its motion control methods in assisting surgeons in performing brachytherapy on the liver tumor, improving its motion safety and easing the use of a robot with US probe. In this paper, we present a 5-DoF robotic system with axial force and US image feedback for radioactive seeds implantation in HCC. The main contributions are as follows: (1) A novel mechanical structure for robot-assisted brachytherapy on the liver tumor is proposed. The robotic system provides surgeons with high-accuracy manipulator and keeps them from the radiation of seeds; (2) APF-based motion planning method is introduced into the multi-DoF robotic system. (3) Safety contact method based on US image was designed for the control strategy, which automates the process of probe contact.

**Fig. 2:**
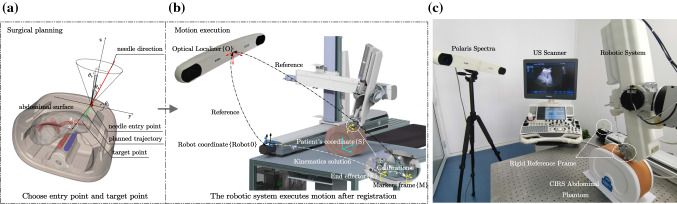
**a**, **b** Workflow of the robotic system in an operation on abdomen phantom; **c** Experiment Setup

## Material and method

### Interventional surgical system

The prototype interventional system consists of two parts: preoperative CT image-based surgical planning and intraoperative robot-assisted seeds implantation, as illustrated in Fig. 2. The operating workflow is as follows:

*Surgical planning* The process of surgical planning is executed manually by the operator. First, surgeons attach several markers to the surface of the abdomen for image registration before CT scanning. Second, with the scanning images, tumor segmentation and 3D reconstruction are done by 3D Slicer. Third, to implant radioactive seeds into the tumor, radiologists define the distribution of the seeds and decide the needle entry points and target points in the model. The needle path is determined by the two sets of points from the skin to the tumor in CT 3D reconstruction space.

*Motion execution* Before the system performs the motion, surgeons should complete the registration between the patient and the space of OTS. After the registration, the process of motion planning and execution (until the probe contacts with the surface) is automatically implemented with the methods in Sect. 2.3: First, the system can complete the registration of robot space by the passive markers on it (Experiment setup in Fig. 2). It will find the trajectory to the goal of motion planning by the APF-based method in Sect. 2.3.1 and move to the goal along the trajectory. Then, the end effector approaches the surface until the probe fully contacts with it, where the safety contact method is described in Sect. 2.3.2. Finally, the operator can adjust the pose of the end effector and perform insertion by the provided GUI (Fig. 9) under the monitor of real-time US image.

### Design of seeds implantation robot

#### Mechanism design

**Fig. 3:**
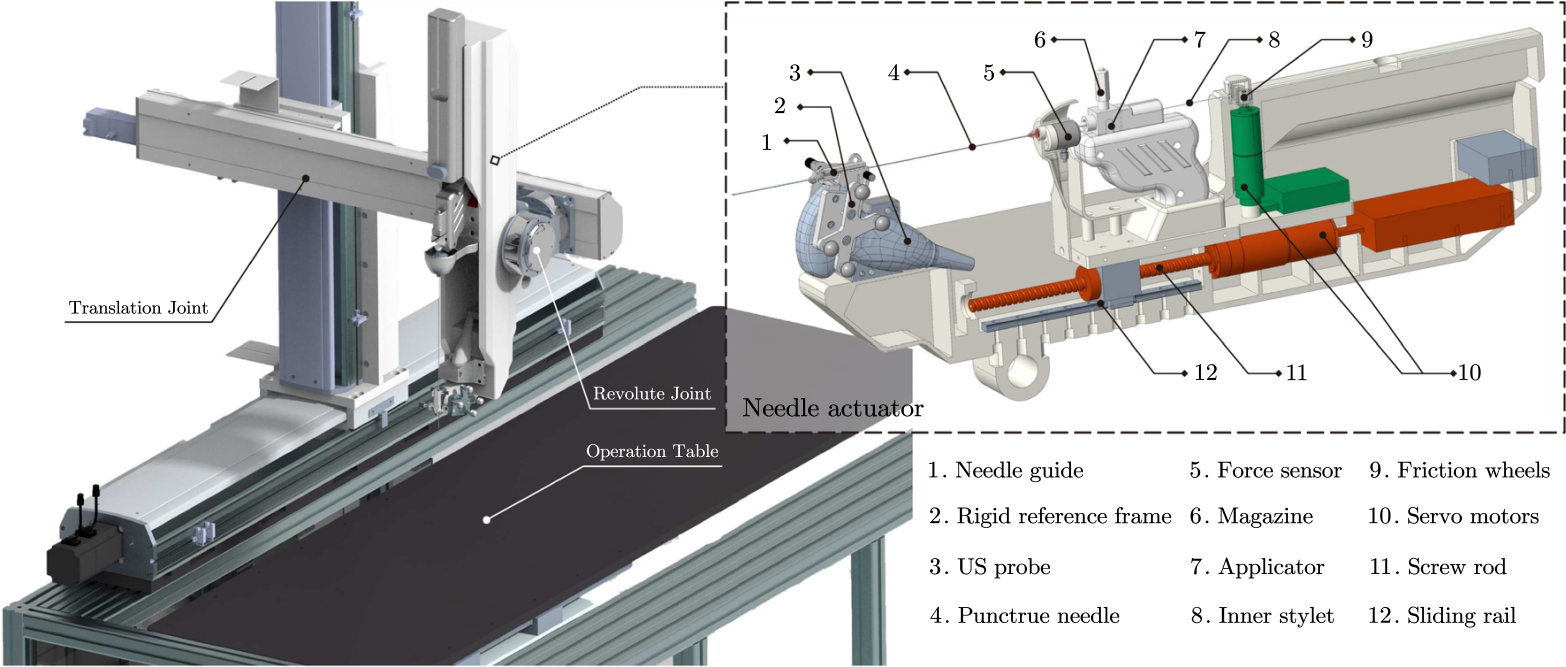
Mechanism overview of the robotic system

The proposed robotic system is comprised of a three-axis slide module, a two-axis revolute joint and a needle actuator (Fig. 3). The needle actuator is equipped with an axial force sensor, a US probe and a seed implantation module, as is shown on the left side of the figure.

The three-axis translation joint and two-axis revolute joint ensure that the system has enough DoF to meet the need of the workspace. The translation joint is driven by Delta Servo System (ASDA-B2 Series, Delta Electronics, China), providing the robotic system with a large workspace to place the end effector ($527.5\times 750 \times 603.4$ mm$^3$). The revolute joint is driven by two servo motors (HT-03, Hai Tai Electromechanical Equipment, China) with a high continuous torque of 6.9NM. Together with the translation joint, which has a maximum loading of 15 kg on the 3rd arm, the high continuous torque involves a redundant capability of driving the needle actuator, making high-load and long-time running of the robotic system possible.

The needle actuator is comprised of multiple components and is driven by a two-DoF Maxon Servo System (Maxon motor AG, Switzerland), one for needle insertion and the other for seeds implantation. For needle insertion, the linear motion is driven by motorized screw and sliding rail, ensuring the stable and continuous control of insertion. The needle is fixed to a disposable part attached to the force sensor (SBT671, SIMBATOUCH, China), which can measure the axial force experienced by the needle in operation. The inner stylet is driven by a pair of friction wheels made of silicon. The friction wheels are also aseptic and disposable, which can be replaced after finishing operation. A rigid reference frame is fixed on the needle guide for calibration, which will be further discussed in Sect. 2.2.3.

#### Forward kinematics

**Fig. 4:**
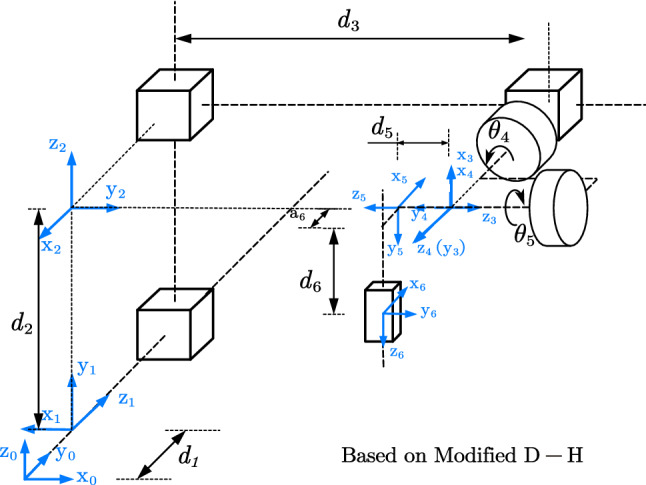
Schematic representation of robot’s kinematics

Figure 4 is a schematic of the kinematic model of the robotic system, where the end effector is the tip of the needle. The model is comprised of four prismatic joints and two revolute joints, except for the seeds implantation joint. Link parameters are assigned based on the modified Denavit-Hartenberg convention, which is shown in Table 1.

The transformation matrix of the end effector regarding the base of the robot can be obtained by the homogenous matrix of each link. In particular, $^\mathrm{{Robot0}} T_\mathrm{{Robot5}}$ (Eq. 1) is shown separately for the need of computation scheme, which will be further discussed in Sect. 2.2.3.

1 $$\begin{aligned} \begin{array}{llll} ^\mathrm{{Robot0}} T_\mathrm{{Robot5}}=\begin{bmatrix} -c{\theta _5}'s\theta _4 &{}\quad s\theta _4s{\theta _5}' &{}\quad -c\theta _4 &{}\quad d_3-d_5\cdot c\theta _4 \\ s{\theta _5}' &{}\quad c{\theta _5}' &{}0 &{}d_1 \\ c\theta _4 c{\theta _5}' &{}\quad -c\theta _4 s{\theta _5}' &{}\quad -s\theta _4 &{}\quad d_2-d_5\cdot s\theta _4 \\ 0 &{}\quad 0 &{}\quad 0 &{}\quad 1 \end{bmatrix} \end{array} \end{aligned}$$

where ${\theta _5}' = \theta _5 + 90^\circ$, $c = cos()$, $s=sin()$, {Robot i} means the coordinate system of joint *i*. $^\mathrm{{Robot0}} T_\mathrm{{Robot5}}$ means the transformation matrix of {Robot5} with respect to {Robot0}.

#### Inverse kinematics

The system can be described under 6 coordinate systems: The optical positioning coordinate system {O}, the 3D phantom model’s coordinate system {C}, robotic base coordinate system {Robot0}, the end effector coordinate system {E}, the passive markers frame on US probe {M} and patient’s coordinate system {S}.

**Fig. 5:**
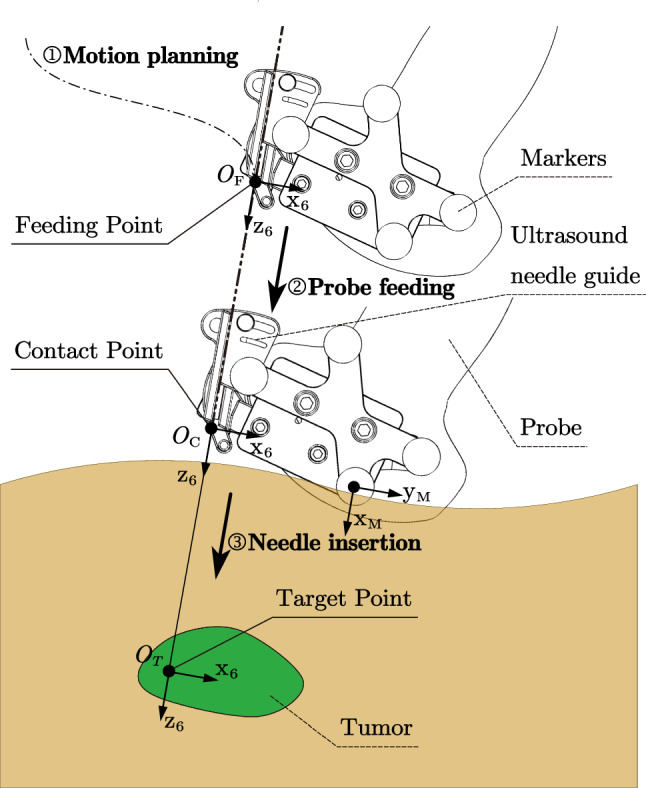
Control strategy:{E} first arrives at Feeding point, then moves along the inserting orientation, finally reaches contact point and inserts needle

In robot space, the transformation matrix $^\mathrm{{Robot0}} T_T$ can be calculated for a given target $P_T (x_T, y_T, z_T)$ and its orientation $({\theta _4}_T, {\theta _5}_T)$. Motion planning uses only 5-DoF of the robotic system (without the DoF used for insertion and seed implantation). Therefore, we defined a fixed point E as the end effector at the exit of the needle guide, which is shown up as $O_F$ and $O_C$ in Fig. 5 and {E} in Fig. 2. As for the fixed point, it can be regarded as an offset from Joint 5 to Joint 6 and simplifies the path planning computation. Variables $d_1, d_2, d_3, \theta _4, \theta _5$ can be obtained based on Eq. (2):

2 $$\begin{aligned} ^\mathrm{{Robot0}} T_T (^\mathrm{{Robot5}} T_E)^{-1} = ^\mathrm{{Robot0}}T_\mathrm{{Robot5}} \end{aligned}$$

where $^\mathrm{{Robot0}} T_T$ is the transformation matrix of the given target relative to {Robot0}. $^\mathrm{{Robot5}} T_E$ is a constant matrix.

When it’s in {O}, the matrix $^O T_{T}$ can be obtained by giving a target point and entry point like FP and CP (Fig. 5). Then the variables can be deduced as shown in Eq. (3):

3 $$\begin{aligned} (^OT_\mathrm{{Robot0}})^{-1} {^{O}T_{FP}} {(^\mathrm{{Robot5}}T_E)}^{-1} = ^\mathrm{{Robot0}}T_\mathrm{{Robot5}} \end{aligned}$$

where $^{O}T_\mathrm{{Robot0}}$ represents the transformation matrix between the coordinate system of {Robot0} to {O}.

To obtain the pose transformation matrix $^O T_\mathrm{{Robot0}}$, a passive rigid reference frame is fixed on the body of the first translational joint, thus the matrix $^O T_\mathrm{{Robot0}}$ can be measured directly.

**Table 1:** D-H parameters of each joint

i	$a_{i-1}$	$\alpha _{i-1}$	$d_i$	$\theta _i$
1	0	$-$ 90	$d_1$	180
2	0	$-$ 90	$d_2$	90
3	0	$-$ 90	$d_3$	$-$ 90
4	0	$-$ 90	0	$\theta _4$
5	0	$-$ 90	$d_5$	$\theta _5$ + 90
6	$a_6$	$-$ 90	$d_6$	0

Considering the deviation caused by deformation of the mechanism, a frame with passive markers was attached to the needle-guide. With the frame, the OTS can measure the real-time pose matrix $^O T_M$ of the frame {M} relative to {O}, which can in turn act as feedback to calibrate the robotic system. When the distance between the feedback and the expected position larger than 1 mm, the error will be calculated and the robot will try to reduce the error until the distance is less than 1 mm.

### Motion control of the robotic system

To further ensure the safety of robot’s motion, we designed a control strategy in Fig. 5, where the figure shows three steps of the motion: (1) {E} is initialized at the original position, and move to the feeding point (FP) by the motion planning algorithm. (2) After reaching FP, the robotic system will feed the probe along with the expected direction, until it arrives at the contact point (CP), which means the probe fully contacts with the skin surface. (3) Finally, after the confirmation of the operator, the system starts inserting under the monitoring of US image and force feedback. The process of insertion can also be performed by the operator, who can adjust the pose of the end effector by GUI before insertion (Fig. 9).

CP is the position where the probe contacts sufficiently with the skin, suggesting that the system is able to acquire high-quality US images. FP is a point above CP along the needle direction (where $O_F,O_C,O_T$ locate in the same line), which is the goal of motion planning. It is decided by determining the length of $|O_TO_F|$.

#### Robot motion planning method based on APF

Since the operation of brachytherapy for the liver tumor requires US image to monitor the needle and tissue under the skin, a robot with a US probe will move close to the patients and contact with them. Therefore, the movement should avoid physical injury as well as collision with the environment [5], such as probe rubbing and colliding. We introduced APF-based approach [11] into the process of motion planning.

**Fig. 6:**
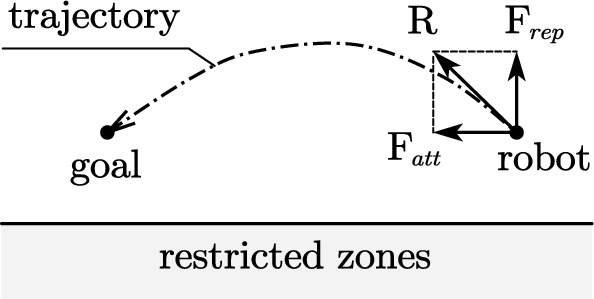
Free body diagram of the robot in APF, where the restricted zones are sets of points

**Fig. 7:**
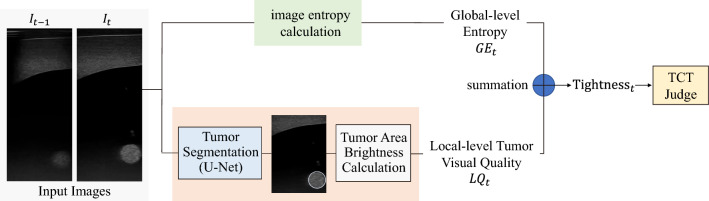
Composition of the Probe-Tissue Contact Detection module

In application, the point cloud, generated by 3D reconstruction of the patent’s pre-operative CT-image, is defined as restricted zones (Fig. 6) relative to the robotic system in this method. Repulsive field or force is calculated from a single “collision point”. Every single point on the surface of this point cloud contributes to the motion planning of the robot. To simplify the calculation, only points within a specific radius of the end effector are involved in the planning process.

Figure 6 shows the principle of APF, where attractive force ${\varvec{F}}_{att}({\varvec{q}})$ from goal and repulsive force ${\varvec{F}}_{rep}({\varvec{q}})$ from restricted zones are imposed on the robot (the end effector). With resultant force *R* of the two force, the robot will move towards the goal while moving away from the restricted zones. Consequently, the robot will move to the goal in a trajectory without colliding with the restricted zones. The attractive potential function $U_{att}({\varvec{q}})$ and attractive force ${\varvec{F}}_{att}({\varvec{q}})$ are shown on Eq. (4) and (5).

4 $$\begin{aligned} U_\mathrm{{att}}({\varvec{q}})&= \frac{1}{2}\varepsilon \rho ^2 ({\varvec{q}},{\varvec{q}}_\mathrm{{goal}}) \end{aligned}$$

5 $$\begin{aligned} {\varvec{F}}_\mathrm{{att}}({\varvec{q}})&= -\nabla U_\mathrm{{att}}({\varvec{q}})=\varepsilon ({\varvec{q}}_\mathrm{{goal}} - {\varvec{q}}) \end{aligned}$$

where $\varepsilon$ is a positive scaling factor, and $\rho ({\varvec{q}},{\varvec{q}}_\mathrm{{goal}})$ is the distance between the end effector and the goal.

As for the planning task in this system, the goal is always close to the restricted zones, which will bring about goals non-reachable problem. Therefore, we used an improved repulsive potential function $U_\mathrm{{rep}}({\varvec{q}})$ proposed in [11], as shown in Eq. (6).

6 $$\begin{aligned} U_\mathrm{{rep}}(\mathbf{q} )= {\left\{ \begin{array}{ll} \frac{1}{2}\eta (\frac{1}{\rho ({\varvec{q}}\cdot {\varvec{q}}_\mathrm{{obs}})}-\frac{1}{\rho _0})^2 \rho ^n ({\varvec{q}}\cdot {\varvec{q}}_\mathrm{{goal}}) &{} \text {if}\, \rho ({\varvec{q}}\cdot {\varvec{q}}_\mathrm{{obs}}) \le \rho _0\\ 0 &{} \text {if}\, \rho ({\varvec{q}}\cdot {\varvec{q}}_\mathrm{{obs}}) > \rho _0 \end{array}\right. } \end{aligned}$$

where $\eta$ is a positive scaling factor, $\rho _0$ is a positive constant denoting the distance of influence of the obstacle (a single collision point in the point cloud). The corresponding repulsive force ${\varvec{F}}_\mathrm{{rep}}({\varvec{q}})$ of a single collision point is given by

$$\begin{aligned} {\varvec{F}}_{rep}({\varvec{q}})&=-\nabla U_{rep}({\varvec{q}}) \\&= {\left\{ \begin{array}{ll} F_\mathrm{{rep1}}{\varvec{n}}_\mathrm{{OR}} + F_\mathrm{{rep2}}{\varvec{n}}_\mathrm{{RG}} &{} \text {if}\, \rho ({\varvec{q}}\cdot {\varvec{q}}_\mathrm{{obs}}) \le \rho _0 \\ 0 &{} \text {if}\, \rho ({\varvec{q}}\cdot {\varvec{q}}_\mathrm{{obs}}) > \rho _0 \end{array}\right. } \end{aligned}$$

where

7 $$\begin{aligned} F_{rep1}&= \eta (\frac{1}{\rho ({\varvec{q}}\cdot {\varvec{q}}_\mathrm{{obs}})}-\frac{1}{\rho _0}) \frac{\rho ^n({\varvec{q}}\cdot {\varvec{q}}_\mathrm{{goal}})}{\rho ^2({\varvec{q}}\cdot {\varvec{q}}_\mathrm{{obs}})} \end{aligned}$$

8 $$\begin{aligned} F_{rep2}&= \frac{n}{2} \eta (\frac{1}{\rho ({\varvec{q}}\cdot {\varvec{q}}_\mathrm{{obs}})}-\frac{1}{\rho _0})^2 \rho ^{n-1}({\varvec{q}}\cdot {\varvec{q}}_\mathrm{{goal}}) \end{aligned}$$

${\varvec{n}}_\mathrm{{OR}}=\nabla \rho ({\varvec{q}},{\varvec{q}}_\mathrm{{obs}})$ and $\mathbf{n} _\mathrm{{OR}}=-\nabla \rho (\mathbf{q} ,\mathbf{q} _\mathrm{{goal}})$ are two unit vectors pointing from the obstacle to the end effector and from the robot to the goal, respectively. The net repulsive force is calculated as a sum of repulsive force from each collision point within a specific radius of the end effector.

#### Safety contacting method based on real-time US image

Based on the trajectory provided by the motion planning step, the end effector reaches FP and starts to approach the surface until the probe contacts with it and obtains US images. Since the robot needs the maximum visual quality of tumors to perform precise brachytherapy operation, high-quality US images are preferred as visual feedback to the operator. However, high-quality US images can only be obtained when the probe contacts the skin tightly enough. The time and the location where the pose satisfies the condition is defined as Tight Contact Timestamp (TCT) and CP.

To determine the TCT and CP during the procedure of probe contacting with the skin, a US image-based safety contact method is developed. This method takes real-time US images acquired from the probe as input and decides which timestamp of the acquired image is TCT. Once the TCT is found, the robot would stop advancing, suggesting that it reaches CP. The safety contacting method comprised of three parts, namely entropy calculation, U-Net [12] based local brightness calculation and TCT judge, as illustrated in Fig. 7.

The $\hbox {Tightness}_t$ is the summation of global-level entropy and local-level tumor visual quality for the US image $I_t$ acquired at timestamp(*t*). The global-level entropy $\mathrm {GE}_t$ is a statistical measurement of randomness that can be used to characterize the texture of the input grayscale image. The entropy is positively associated with the richness of the information in US images.

9 $$\begin{aligned} \mathrm {GE}_t = -\sum _{i=1}^{M\cdot N}p_i \log (p_i) \end{aligned}$$

$p_i$ is occurrence probability of the intensity level *i* in the histogram of $I_t$ $0\le p_i \le 255$

The U-net, a commonly used neural network architecture, takes each US image as input and segment the tumor in US images after being trained based on an US-image dataset on tumors (containing over 1000+ images), which we collected and annotated pre-operatively. The segmented tumor area is defined as $R_t$, from which we calculate the local-level tumor visual quality $LQ_t$ is calculated based on Eq. (10) and (11).

10 $$\begin{aligned} \mathrm {LQ}_t&= \frac{\sum _{i=0}^{255}|i-128-\mathrm{{da}}|\cdot \mathrm{{hist}}(i)}{\mathrm{{area}}(R_t)} \cdot (\mathrm {da}+128) \end{aligned}$$

11 $$\begin{aligned} \mathrm{{where~da}}&= \frac{\sum _{(m,n)\in R_t}(I_{m,n} - 128)}{\mathrm{{area}}(R_t)} \end{aligned}$$

$I_{m,n}$ is the intensity at position (*m*, *n*) insides $I_t$. The position (*m*, *n*) only lies inside the tumor area $R_t$. $\mathrm{{hist}}(i)$ is the number of occurrences of the intensity level *i* in the histogram of $R_t$. $area(R_t)$ is defined as the size of $R_t$.

**Fig. 8:**
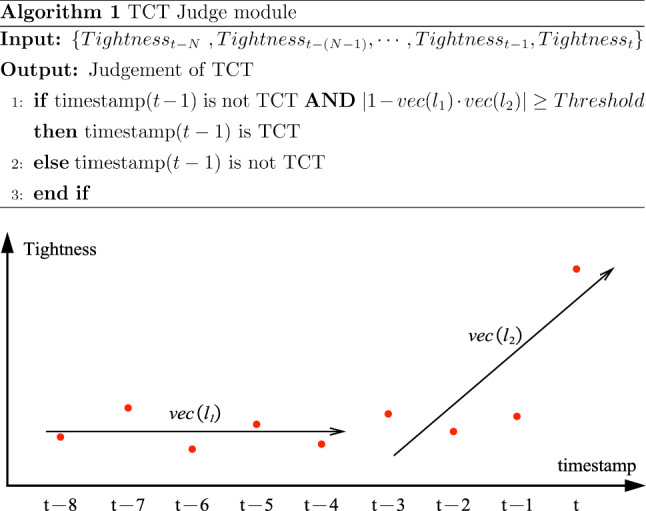
TCT Judge module and illustration of the stopping criteria

Equations (10) and (11) are used for calculate “brightness” and “contrast” for input image, respectively. *da* in Eq. (11) is defined as the mean value of pixel intensity inside the tumor area. The former summation part in Eq. (10) is defined as the arithmetic average deviation of pixel intensity inside the tumor area. Ideally, if the probe touches the tissue more tightly, then the tumor area should be brighter and the da should be larger. Meanwhile, the contrastness of tumor area should be greater, and the average deviation should be larger. As the texture inside the tumor area changes more significantly (than the background area) in the probe contacting process, the two equation are adapted to calculate the area rather than global image.

In this contact process, multiple US images and the corresponding tightness are obtained consecutively. At timestamp(*t*), the TCT Judge module takes the tightness $\hbox {Tightness}_t$ and tightness of previous *N* frames $\{\hbox {Tightness}_{t-N}$ $, \hbox {Tightness}_{t-(N-1)},\cdots , Tightness_{t-1}\}$ as input and determines whether timestamp(*t*) is TCT, or the end effector locates at CP, as described by the pseudo-code in Fig. 8.

The plot in Fig. 8 illustrates how $l_1$ and $l_2$ linearly regress on {$(t-N, \hbox {Tightness}_{t-N}),\cdots ,(t-N/2, \hbox {Tightness}_{t-N/2})$} and {$(t-N/2, \hbox {Tightness}_{t-N/2}),$ $\cdots ,(t, Tightness_t)$}. $vec(l_i)$ denotes the directional vector of the regressed line $l_i$. Ideally, when timestamp(t) is TCT and the probe starts to touch the tissue tightly, the tightness at timestamp(*t*) should change abruptly than timestamp($t - 1$). This abrupt change can be translated, in terms of math, into the judgement that the angle between two normalized vector $vec(l_1)$ and $vec(l_2)$ is large enough.

**Fig. 9:**
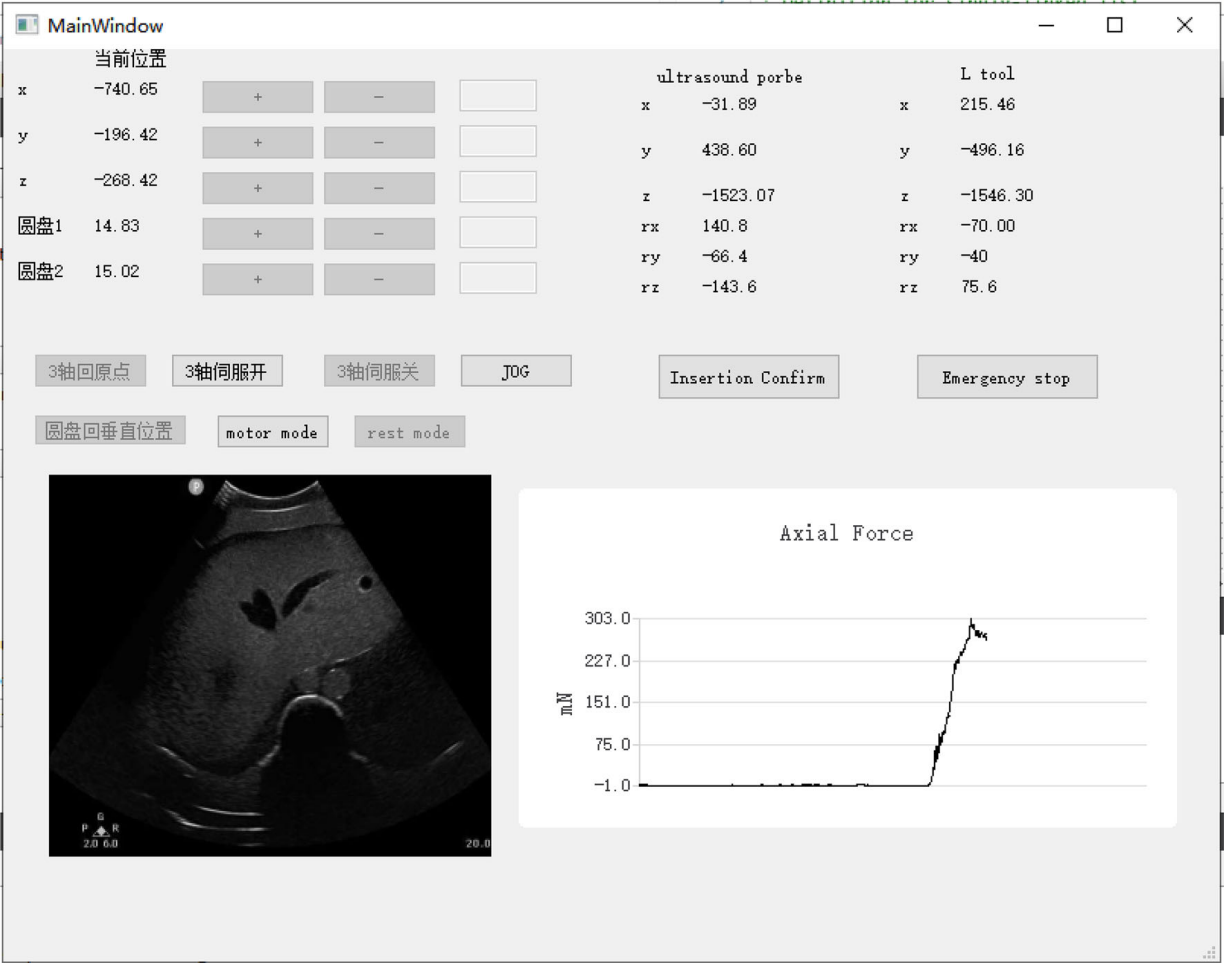
Design of the console

Considering the deformation and shift of the liver in the abdomen, which may be caused by breathing or intra-operative movement, we also provide the operator with a console to adjust the robot manually (Fig. 9). The operator can manipulate the robot with the real-time feedback of US image and axial force.

## Experiment result

We conducted three kinds of experiments to evaluate the designed robot and validate the aforementioned methods. First, to find out the position and orientation accuracy about {E}, a series of targeting experiments with OTS were performed. Next, to evaluate the performance of the motion control strategy, we conducted an abdominal phantom based experiment and tested the algorithm’s performance in different situations by simulation. The performance of contact method is also illustrated by an example experiment result. At last, we analyzed the axial feedback force in a typical insertion experiment.

### Accuracy evaluating experiment

As the process of needle placement was performed by physicians in our current framework, the accuracy experiments focus on testing the positioning performance of the system in executing motion, ensuring that the robot can well position at the decided location. Due to the large workspace of ($$527.5\times 750 \times 603.4$$ mm$$^3$$), it is difficult to sample the points throughout it. Therefore, we defined a sub-workspace according to the anthropometric data of adults [13] (Fig. 11a) and sampled 60 points to perform the accuracy evaluation experiments, where 20 points for translation without rotation and 40 points for translation with rotation. The results were shown in Fig. 11b and 11c. The mean of positioning error is 0.69 mm and the standard deviation is 0.33 mm. We calculated the integral of the error along each dimension (Fig. 11**c**). The goodness of fit showed that linear model could be used to explain those curve ($$R^2_x = 0.9818$$, $$ R^2_y = 0.9902$$, $$R^2_z = 0.9959$$, the *p*-value on each axis is less than 0.001 with 0.95 confidence bounds). Therefore, the distribution of the error could be regarded as uniform in the experiment region.

**Fig. 10:**
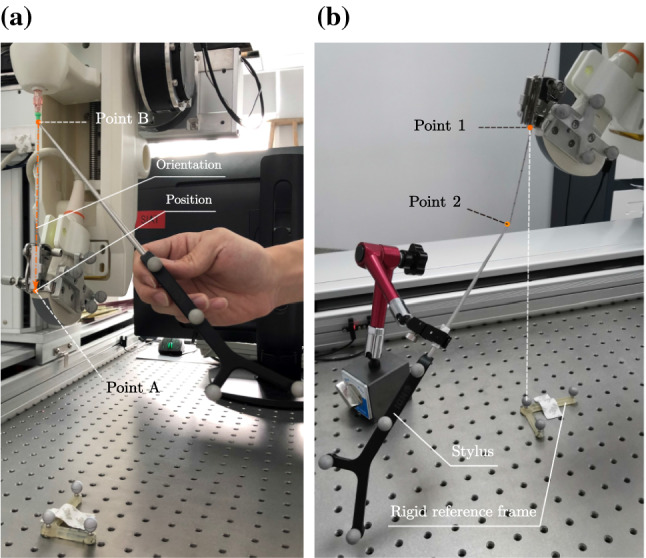
Accuracy evaluating experiments: **a** Two points measured for evaluating the position and orientation of the end effector; **b** The robotic system stopped at Point 1 and inserted its needle to Point 2

We also conducted experiments to evaluate the accuracy of needle insertion (Fig. 10b). Point 1 was defined in the local coordinate system of the fixed rigid reference frame above the optical table, and the other point was defined at the tip of the stylus. The end effector should move to Point 1 and insert its needle to Point 2. After the robotic system finishing the motion, the distance between the needle tip and stylus’s tip was recorded manually. We sampled 10 points for evaluating inserting accuracy. The mean and standard deviation of these points is 1.10 mm and 0.33 mm, respectively.

### Motion control experiment

In this experiment, we used an abdominal phantom (Model 057A, Cirs, USA) with eight markers (#128, Beekley Medical, USA) (Fig. 12).

After CT scanning and 3D reconstruction of the phantom, we selected a target point and an entry point in {C}. Figure 12b shows the 3D model of the phantom. With the method in Sect. 2.2.3, we obtained the relationship between each coordinate system and had a trajectory based on APF planned (Fig. 12d).

The robotic system moved along the trajectory generated by APF (Fig. 12e and 12f), arriving at the feeding point (Fig. 12g), and finally reached the contacting point in the expected orientation (Fig. 12h). The experiment showed that the implementation of pre-operative planning with APF and control strategy can protect the robot from colliding with the registered restricted zones like a phantom.

To further evaluated the algorithm’s performance in different situations, we also conducted simulation experiments in different cases and parameters (Fig. 13). In case (a) and case (d), we can see that, without collision-avoidance algorithm, the robot will collide with restricted zones (dotted line) on its way to the goal. In case (b) and case (c), although the trajectory from the start to the goal is less likely to incur collision, the utilization of APF makes the movement safer (line $\triangle , \blacktriangle , \circ , \blacksquare$), where a larger scale of $\eta$ can make the moving safer and a smaller one can make the moving faster.

**Fig. 11:**
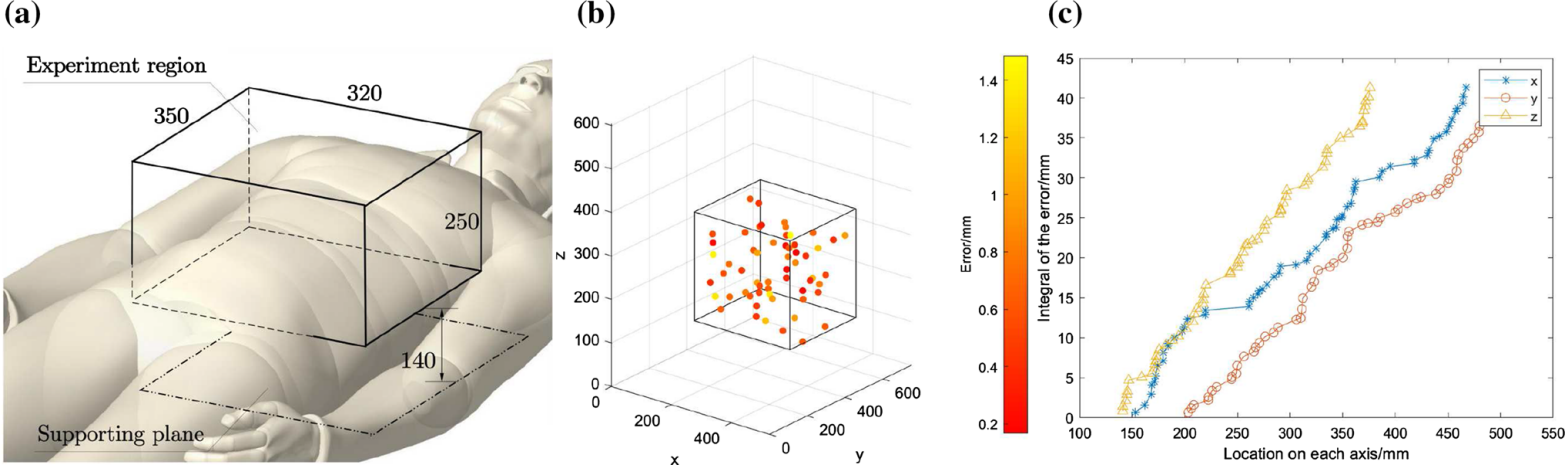
**a** Dimension and location of the experiment region (sub-workspace); **b** Error distribution of the sampled points; **c** Integral of error along each dimension

**Fig. 12:**
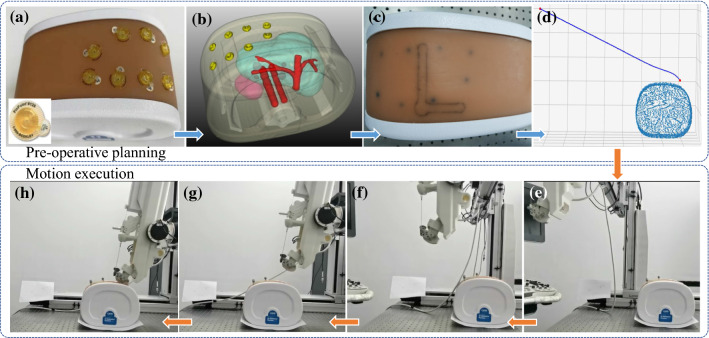
*Pre-operative planning*: **a** Abdominal phantom with eight markers; **b** 3D model of the phantom after CT scanning and reconstruction; **c** Registration point on the phantom; **d** Motion planning by APF after registration; *Motion execution*: **e** On starting point; **f** On the way; **g** On the feeding point; **h** On the contacting point

**Fig. 13:**
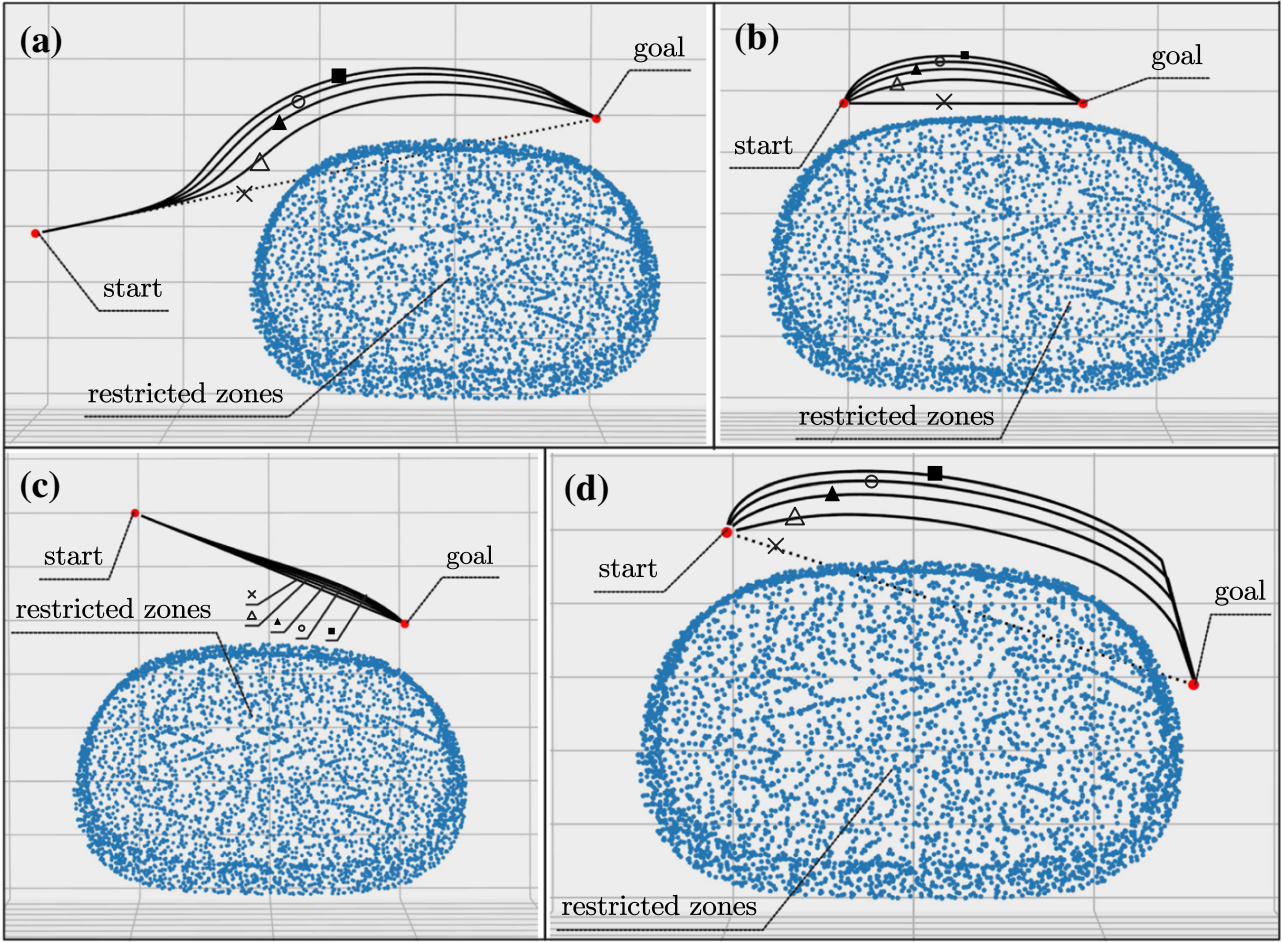
Robot trajectory planning result with APF in different cases, where $\times$ means planning without collision-avoiding algorithm, $\triangle , \blacktriangle , \circ , \blacksquare$ are the results with parameters $(\varepsilon , \eta )$ = (2, 5), (2, 10), (2, 15), (2, 20)

**Fig. 14:**
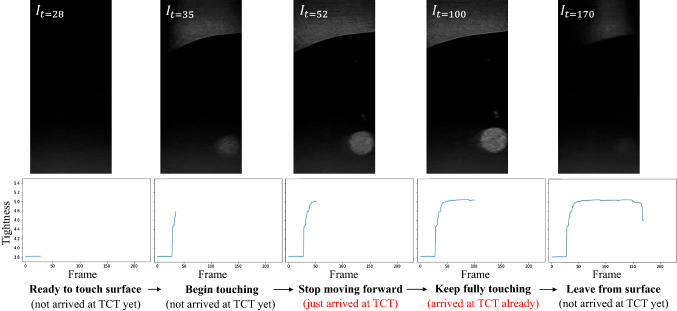
Result of the Probe-Tissue Contact Detection module

An example result of Probe-Tissue Contact Detection is shown in Fig. 14, which illustrates the process of probe contacting with enough ultrasonic couplant. At frame 35, the probe has touched tissue but not tightly yet. As the probe touched the tissue more and more tightly, the visibility of the tumor and the texture of the global image got increased. The component detected that the probe has touched the tissue tightly at frame 52. Therefore, TCT is defined as timestamp 52, which was consistent with the human observation that visual quality of US image at timestamp 52 is maximized. After reaching TCT (timestamp 52), the probe stopped moving forward (timestamp 100). At timestamp 170, the probe was lifted manually by the console and Tightness dropped rapidly as the probe separated from the surface.

### Force evaluation in insertion experiment

**Fig. 15:**
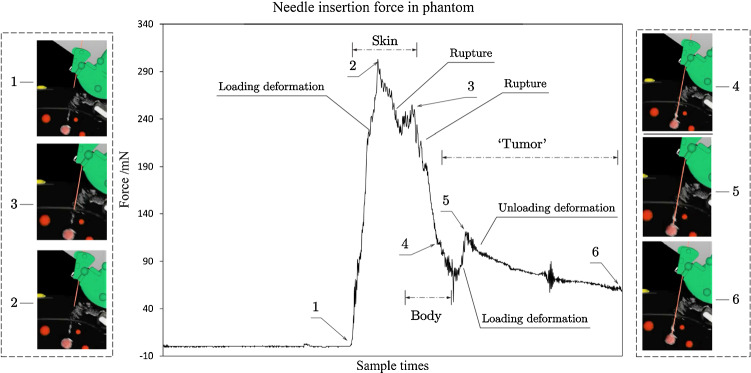
Axial force experienced by needle when inserting in the phantom

According to the force-displacement characteristics in [14], the needle experienced the event of loading deformation when contacting the skin (1$\rightarrow$2) as illustrated in Fig. 15. When the force reached its maximum, a crack suddenly propagated into the skin, causing a rupture event (2$\rightarrow 3$). From 2 to 3, the needle’s tip cut through the skin and got into the body in 3, where also caused a loading deformation and rupture event (3$\rightarrow$4) when contacting body tissue. When the needle reached 4, it encountered the “tumor” in the phantom and experienced the event of loading deformation (4$\rightarrow$5) again. Then the needle stopped at the center of the “tumor”, which made elastic potential energy decrease slowly (5$\rightarrow$6). From 5 to 6, the needle was experiencing the event of unloading deformation.

## Discussion

Radioactive seeds implantation is an effective, minimally invasive treatment method for malignant liver tumors in HCC. For this treatment, robotic systems can provide surgeons with accurate needle insertion and seeds implantation under the guidance of pre-operative CT scanning, and several research groups have proposed their work in robotic system assisted needle insertion and implantation. So far, few works have been proposed about robot-assisted brachytherapy for malignant liver tumors in HCC. In this paper, a 5-DoF robotic system with US image and force feedback is developed for the therapy. The experiment results in Sect. 3.1 show that the proposed structure can position itself accurately by the mean error of 0.69 mm, which meets the requirement of acceptable accuracy of 2 mm. The system accuracy regarding the end effector consists of mechanical errors and optical localizer errors. Those errors can be calibrated by the position feedback of the OTS, making the positioning accuracy less than the decided threshold of 1 mm. Whereas, to measure the positioning error directly, we used stylus in the experiment Fig. 10, which introduced the measuring error to the result in Fig. 11. Due to the measuring error, some of the measurements in Fig. 11b showed excessive deviation, making the error larger than the decided threshold.

In the procedure of motion planning, the performance of the method was tested in Sect. 3.2. The results show that it makes the movement of robotic system safer in some common cases in Fig. 13, which are some typical planning tasks at the risk of collision and rubbing between the probe and the skin. This paper extends the research by Zhu et al. and Zequn et al. and introduces the APF-based approach into the planning. Comparing to their works, the method utilizes the registered model for collision-avoidance path planning, which does not increase the complexity or setup times of the operation while making the motion of robot safer. The collision-avoiding method in this paper is for static objects, which have been well registered to {O} before operating. For dynamic objects like the breathing abdomen and surgeons as well as other unstructured items, the method can be further developed by using Kinect (Microsoft, USA), which has shown effectiveness in active collision avoidance [15].

Besides, for the underlying problem with the shift of the liver caused by breathing, we mounted the US probe on the robot and provided the surgeon with US image feedback. As the probe can obtain high-quality US images only if it touches tightly with the skin, we developed a contacting method for the control. The example result in Fig. 14 validates the feasibility of the method in detecting the tightness between the probe and the skin. In the experiments, we used enough ultrasonic couplant to ensure high image quality during the contacting process, which made it possible to acquire optimized quality soon after contacting with the surface instead of increasing pressure on the probe. It also makes the change on the computing result of detecting module significant. Without enough ultrasonic couplant, the probe may be pushed further and cause excessive pressure on the surface. Contact force in the contact process is unavailable for the current robotic system. Considering the potential risk of excessive force, we will embed an axial force sensor separately for the probe in the next generation of the robot. With the help of US images and the measured force, sufficient contact can be obtained while avoiding excessive force to cause injury to the patient.

The information from force feedback and US image hasn’t been utilized sufficiently for the control system. We are trying to make full use of the information provided by the US image. For example, respiratory signal can be extracted from intraoperative US image sequence, which can be useful to determine the optimal needle insertion moment. By integrating our previous work on US probe spatial calibration method [16], we can also obtain the spatial position and orientation of the real-time 2D US image. However, adjusting the direction of insertion is unavailable with the current robot. There requires another DoF for rotating the needle. We have designed a new needle actuator which can rotate the needle and are planning to perform phantom experiments with the new actuator under the guide of US image in our future work.

## Conclusion

In this paper, a 5-DoF robotic system with force and US image feedback was developed for the brachytherapy of liver tumor. The robotic system consists of a 5-DoF actuator, an US scanner and OTS. The proposed control methods take the safety into consideration, automating the process of motion execution and probe contacting, easing the use of the robotic system. After the end effector is in place, the operator can adjust the robot by the provided GUI with the US image and force feedback. In future work, we will continue increasing the accuracy and safety of the robotic system by improving its mechanism design and image-based methods. Besides, we will also perform phantoms and animal experiments to verify the efficacy and accuracy of the system.
